# Supplementary material for: Plant defences mediate interactions between herbivory and the direct foliar uptake of atmospheric reactive nitrogen
Source: Nat Commun. 2018 Nov 9;9:4743. doi: 10.1038/s41467-018-07134-9 (PMC6226520; doi:10.1038/s41467-018-07134-9)
Supplement: Supplementary file 1 — Supplementary Information [file 41467_2018_7134_MOESM1_ESM.pdf]

# Supplementary Information

Plant defences mediate interactions between herbivory and the direct foliar uptake of atmospheric reactive nitrogen

Campbell *et al.*

**Contents**

Supplementary Figure 1: Correlations between amount of NO2-derived N and foliar metabolites.

Supplementary Figure 2: Biomass, C:N, %N of plants

Supplementary Figure 3: Effects of NO2 exposure and herbivory on foliar %N (experiment 2)

Supplementary Table 1: Herbivore performance statistical results (main experiment)

Supplementary Table 2: Metabolomics statistical results (main experiment)

Supplementary Table 3: Plant morphological data statistical results (main experiment)

Supplementary Table 4: Foliar uptake statistical results (main experiment)

Supplementary Table 5: Plant C:N and %N statistical results (main experiment)

Supplementary Table 6: Herbivore-induced foliar uptake statistical results (experiment 2)

Supplementary Table 7: Means and standard errors for $^{15}NO_2$ derived N uptake (experiment 1)

## Supplementary Figures

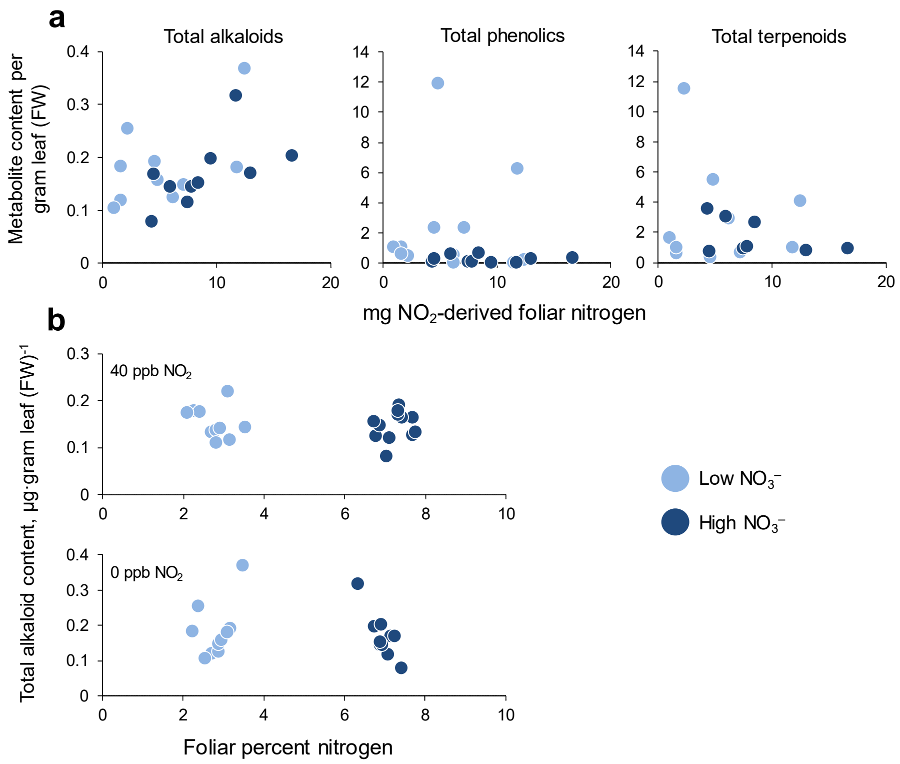


**Supplementary Figure 1. Relationship between defensive metabolites and $NO_2$-derived nitrogen**. **(a)** Pearson correlations between leaf uptake of $NO_2$-derived nitrogen and total alkaloids (r = 0.4587; *P* = 0.0365), phenolics (r = -0.3424; *P* = 0.1188) and terpenoid glycosides (r = 0.2956; *P* = 0.1933) in individual plants under 40ppb $NO_2$. Light blue points are for "Low" (50mM) and dark blue points are for "High" (500mM) root $NO_3$ availability. Alkaloid and phenolic quantities are given as µg·gFW$^{-1}$; terpenoid glycosides are given as peak-area·gFW$^{-1}$. (**b)** Correlations between total alkaloid content and foliar %N at 40ppb $NO_2$ (r = -0.1566; *P* = 0.4980) and 0ppb $NO_2$ (r = -0.1173; *P* = 0.6031).

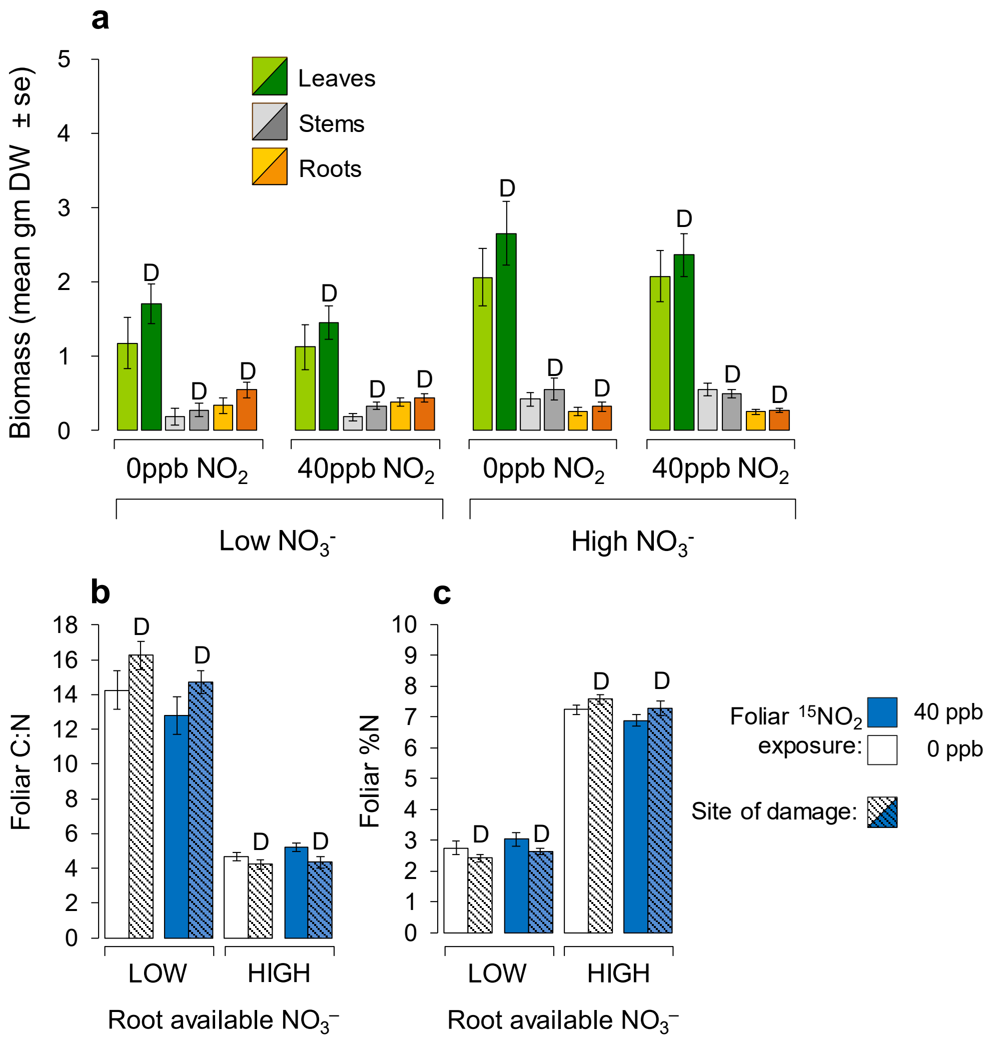


**Supplementary Figure 2. Plant growth and leaf nitrogen results (experiment 1).** Effects of root N availability ("Low", 50mM vs. "High", 500mM $NO_3$), $NO_2$ exposure (0ppb vs. 40ppb), and herbivory (presence vs. absence) on: **(a)** plant biomass components (leaf, root and stem dry weights, DW); **(b)** leaf carbon:nitrogen (C:N) ratios; and **(c)** foliar % N. Herbivore-damaged plants denoted by "D"; hatched bars represent values for damaged leaves in herbivore-exposed plants. Full statistical results provided in Supplementary Tables 3 and 5.

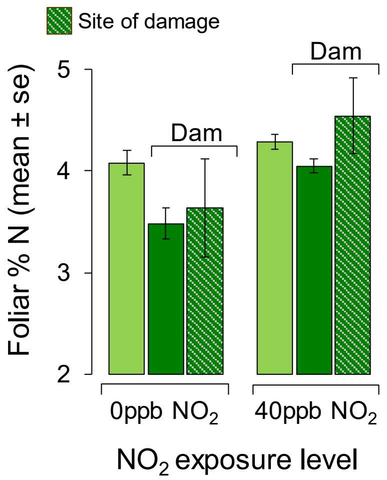


**Supplementary Figure 3. Leaf nitrogen results (experiment 2).** Effect of $NO_2$ exposure (0ppb vs. 40ppb), and prior herbivore-induction (presence vs. absence) on foliar % N. Light green bars are data for control (undamaged) plants; dark green bars are herbivore-exposed plants (denoted by "Dam"); hatching denotes damaged leaves in herbivore-exposed plants. Statistical results provided in Supplementary Table 6.

## Supplementary Tables

**Supplementary Table 1.** Results of linear models testing variation in larval *Manduca sexta* growth and $\delta^{15}N$ after feeding on *Nicotiana tabacum* plants, and larval growth after feeding on artificial diet, as a function of $NO_2$ exposure (40ppb vs. 0ppb), root available $NO_3^-$ (50mM vs. 500mM) and the interaction of $NO_2$ and $NO_3^-$. *P* values < 0.05 in bold.

| | | Source of variation | | |
|---|---|---|---|---|
| Variable | | $NO_2$ | $NO_3^-$ | $NO_2 \times NO_3^-$ |
| Growth on plants | $\chi^2$ | 4.90 | 6.04 | 0.214 |
| | *P* | **0.0266** | **0.0140** | 0.6435 |
| Plant-fed larval $\delta^{15}N$ | $\chi^2$ | 61.09 | 5.07 | 3.84 |
| | *P* | **<0.0001** | **0.0243** | 0.0502 |
| Growth on diet | $\chi^2$ | 0.011 | 3.740 | 0.333 |
| | *P* | 0.9171 | 0.0531 | 0.5640 |

[1]Numerator degrees of freedom (d.f.) = 1 for each model term; error d.f. = 16

**Supplementary Table 2.** Results of linear models testing variation in quantities of defence-related secondary metabolites in *Nicotiana tabacum* leaves as a function of $NO_2$ exposure (40ppb vs. 0ppb), root available $NO_3^-$ (50mM vs. 500mM) and the interaction of $NO_2$ and $NO_3^-$. Labels of individual metabolites correspond to the compounds illustrated in Figure 2. *P* values < 0.05 in bold.

| | | | Source of variation | | |
|---|---|---|---|---|---|
| Metabolite | Label | | $NO_2$ | $NO_3^-$ | $NO_2 \times NO_3^-$ |
| Nicotine | a | $\chi^2$ | 4.0351 | 0.0115 | 1.0094 |
| | | *P* | **0.0446** | 0.9144 | 0.3151 |
| Anatabine | b | $\chi^2$ | 3.9480 | 5.7273 | 2.4798 |
| | | *P* | **0.0469** | **0.0167** | 0.1153 |
| alkaloid 3 | c | $\chi^2$ | 4.3574 | 7.1079 | 0.8483 |
| | | *P* | **0.0368** | **0.0077** | 0.3570 |
| chlorogenic acid | d | $\chi^2$ | 0.0086 | 2.8934 | 0.2779 |
| | | *P* | 0.9262 | 0.0889 | 0.5981 |
| caffeic acid 2 | e | $\chi^2$ | 2.8719 | 5.2941 | 1.0832 |
| | | *P* | 0.0901 | **0.0214** | 0.2980 |
| caffeic acid 3 | f | $\chi^2$ | 1.2379 | 5.3685 | 0.9702 |
| | | *P* | 0.2659 | **0.0205** | 0.3246 |
| diterpene glycoside 1 | g | $\chi^2$ | 5.6580 | 13.6316 | 1.6264 |
| | | *P* | **0.0174** | **0.0002** | 0.2022 |
| diterpene glycoside 2 | h | $\chi^2$ | 3.2007 | 0.8796 | 0.5696 |
| | | *P* | 0.0736 | 0.3483 | 0.4504 |
| diterpene glycoside 3 | i | $\chi^2$ | 0.0031 | 8.0748 | 0.0319 |
| | | *P* | 0.9558 | **0.0045** | 0.8582 |
| diterpene glycoside 4 | j | $\chi^2$ | 0.7520 | 4.2077 | 0.7520 |
| | | *P* | 0.3858 | **0.0402** | 0.3858 |
| total alkaloids | | $\chi^2$ | 4.3673 | 1.6632 | 1.3281 |
| | | *P* | **0.0366** | 0.1972 | 0.2491 |

[1]Numerator degrees of freedom (d.f.) = 1 for each model term; error d.f. = 16

**Supplementary Table 3.** Results of linear models testing variation in plant biomass (dry weights), root:shoot ratios and total leaf surface area in *Nicotiana tabacum* as a function of low and high $NO_2$ exposure (40ppb vs. 0ppb), root available $NO_3^-$ (50mM vs. 500mM), herbivory (presence vs. absence) and all interactions. *P* values < 0.05 in bold.

| | | Biomass | | | | | |
|---|---|---|---|---|---|---|---|
| Model term | | Leaf | Stem | Root | Total | Root:Shoot | Leaf Area ($cm^2$) |
| $NO_3^-$ | $\chi^2$ | 17.7973 | 17.3786 | 11.6811 | 11.6313 | 41.8994 | 30.7576 |
| | *P* | **<0.0001** | **<0.0001** | **0.0006** | **0.0006** | **<0.0001** | **<0.0001** |
| $NO_2$ | $\chi^2$ | 0.2936 | 0.2710 | 0.4187 | 0.1081 | 0.4078 | 0.0057 |
| | *P* | 0.5879 | 0.6027 | 0.5176 | 0.7423 | 0.5231 | 0.9396 |
| $NO_3 \times NO_2$ | $\chi^2$ | 0.0143 | 0.0024 | 5.3463e$^{-5}$ | 0.0008 | 0.5553 | 0.0872 |
| | *P* | 0.9049 | 0.9611 | 0.9942 | 0.9777 | 0.4562 | 0.7678 |
| Herbivory | $\chi^2$ | 4.8798 | 1.9712 | 4.5078 | 4.7726 | 2.0699 | 2.8039 |
| | *P* | **0.0272** | 0.1603 | **0.0337** | **0.0289** | 0.1502 | 0.0940 |
| $NO_3$ × Herbivory | $\chi^2$ | 0.0149 | 0.5035 | 1.4499 | 0.1235 | 1.7515 | 0.1578 |
| | *P* | 0.9029 | 0.4780 | 0.2285 | 0.7253 | 0.1857 | 0.6912 |
| $NO_2$ × Herbivory | $\chi^2$ | 0.5875 | 0.3867 | 1.6683 | 0.8007 | 1.4132 | 1.2160 |
| | *P* | 0.4434 | 0.5340 | 0.1965 | 0.3709 | 0.2345 | 0.2702 |
| $NO_3 \times NO_2$ × Herbivory | $\chi^2$ | 0.0006 | 1.2113 | 0.5084 | 0.0203 | 1.6136 | 0.6297 |
| | *P* | 0.9791 | 0.2711 | 0.4758 | 0.8866 | 0.2040 | 0.4275 |

[1]Numerator degrees of freedom (d.f.) = 1 for each model term; error d.f. = 31

**Supplementary Table 4.** Results of linear models testing variation in atmospherically-derived nitrogen ($^{15}N$) incorporated by direct foliar $NO_2$ uptake into *Nicotiana tabacum* leaf, stem and root tissue as a function of low and high $NO_2$ exposure (40ppb vs. 0ppb), root available $NO_3^-$ (50mM vs. 500mM), herbivory (presence vs. absence) and all interactions. Results for leaf tissue include analyses using (a) locally damaged ("Local") leaves, (b) undamaged leaves on damaged plants ("Systemic") and (c) pooled data for local and systemic leaves, for herbivore-exposed plants. *P* values ≤ 0.05 in bold.

| | | mg $NO_2$-derived N | | | | |
|---|---|---|---|---|---|---|
| | | Leaf | | | Root | Stem |
| Model term | | (a) Local | (b) Systemic | (c) Pooled | | |
| $NO_3^-$ | $\chi^2$ | 6.8462 | 7.8103 | 8.1346 | 8.7520 | 6.1191 |
| | *P* | **0.0089** | **0.0052** | **0.0043** | **0.0031** | **0.0134** |
| $NO_2$ | $\chi^2$ | 56.5989 | 65.7876 | 65.6035 | 43.7843 | 50.4382 |
| | *P* | **<0.0001** | **<0.0001** | **<0.0001** | **<0.0001** | **<0.0001** |
| Herbivory | $\chi^2$ | 29.8227 | 1.4474 | 12.0715 | 1.6158 | 1.6483 |
| | *P* | **<0.0001** | 0.2289 | **0.0005** | 0.2037 | 0.1992 |
| $NO_3 \times NO_2$ | $\chi^2$ | 5.6086 | 6.5952 | 6.7680 | 6.9600 | 6.4238 |
| | *P* | **0.0179** | **0.0102** | **0.0093** | **0.0083** | **0.0113** |
| $NO_3$ × Herbivory | $\chi^2$ | 2.5280 | 0.2116 | 1.0165 | 0.0789 | 1.0163 |
| | *P* | 0.1118 | 0.6455 | 0.3133 | 0.7788 | 0.2043 |
| $NO_2$ × Herbivory | $\chi^2$ | 28.5791 | 1.2697 | 11.3545 | 0.4202 | 1.6116 |
| | *P* | **<0.0001** | 0.2598 | **0.0008** | 0.5168 | 0.3277 |
| $NO_3 \times NO_2$ × Herbivory | $\chi^2$ | 2.1179 | 0.1530 | 0.8269 | 0.0006 | 0.9581 |
| | *P* | 0.1456 | 0.6957 | 0.3632 | 0.9810 | 0.3277 |

[1]Numerator degrees of freedom (d.f.) = 1 for each model term; error d.f. = 31

**Supplementary Table 5.** Results of linear models testing variation in %N and C:N ratios of *Nicotiana tabacum* leaf, stem and root tissue as a function of low and high $NO_2$ exposure (40ppb vs. 0ppb), root available $NO_3^-$ (50mM vs. 500mM), herbivory (presence vs. absence) and all interactions. Results for leaf tissue include a analyses using (a) locally damaged ("Local") leaves, (b) undamaged leaves on damaged plants (i.e. "Systemic") and (c) pooled local and systemic leaves, on herbivore-exposed plants. *P* values ≤ 0.05 in bold.

| | | %N | | | | | C:N | | | | |
|---|---|---|---|---|---|---|---|---|---|---|---|
| | | Leaf | | | Root | Stem | Leaf | | | Root | Stem |
| Model term | | (a) Local | (b) Systemic | (c) Pooled | | | (a) Local | (b) Systemic | (c) Pooled | | |
| $NO_3^-$ | $\chi^2$ | 157.3832 | 150.1848 | 165.1560 | 76.7995 | 91.2110 | 107.1969 | 99.7921 | 108.9820 | 28.6094 | 68.8949 |
| | *P* | **<0.0001** | **<0.0001** | **<0.0001** | **<0.0001** | **<0.0001** | **<0.0001** | **<0.0001** | **<0.0001** | **<0.0001** | **<0.0001** |
| $NO_2$ | $\chi^2$ | 0.1086 | 0.2972 | 0.1114 | 5.2141 | 5.1185 | 1.5447 | 0.7451 | 1.4095 | 1.9296 | 1.7627 |
| | *P* | 0.7417 | 0.5856 | 0.7386 | **0.0224** | **0.0237** | 0.2139 | 0.3880 | 0.2351 | 0.1648 | 0.1843 |
| Herbivory | $\chi^2$ | 0.0077 | 0.2074 | 0.0217 | 5.9261 | 6.5218 | 1.9227 | 0.1084 | 0.4468 | 4.1664 | 5.4105 |
| | *P* | 0.9299 | 0.6488 | 0.8828 | **0.0149** | **0.0107** | 0.1656 | 0.7419 | 0.5039 | **0.0412** | **0.0200** |
| $NO_3^- \times NO_2$ | $\chi^2$ | 5.3504 | 4.7686 | 5.5952 | 5.5584 | 3.3227 | 3.6982 | 3.6332 | 3.8482 | 4.4079 | 1.8797 |
| | *P* | **0.0207** | **0.0290** | **0.0180** | **0.0184** | 0.0683 | **0.0545** | **0.0566** | **0.0498** | **0.0358** | 0.1704 |
| $NO_3^-$ × Herbivory | $\chi^2$ | 8.4831 | 1.0013 | 0.9837 | 3.1163 | 1.8894 | 7.1899 | 0.7687 | 1.0379 | 2.0260 | 0.1156 |
| | *P* | **0.0036** | 0.3170 | 0.3213 | 0.0775 | 0.1693 | **0.0073** | 0.3806 | 0.3083 | 0.1546 | 0.7339 |
| $NO_2$ × Herbivory | $\chi^2$ | 8.60e-5 | 0.0423 | 0.0023 | 3.6271 | 0.9826 | 0.0715 | 0.0408 | 0.0122 | 2.4696 | 0.2401 |
| | *P* | 0.9926 | 0.8370 | 0.9622 | **0.0568** | 0.3216 | 0.7892 | 0.8399 | 0.9122 | 0.1161 | 0.6242 |
| $NO_3^- \times NO_2$ × Herbivory | $\chi^2$ | 0.0758 | 0.1822 | 0.3035 | 5.9417 | 3.9706 | 0.0382 | 0.1560 | 0.1398 | 4.4669 | 5.1787 |
| | *P* | 0.7831 | 0.6696 | 0.5817 | **0.0148** | **0.0463** | 0.8450 | 0.6929 | 0.7085 | **0.0346** | **0.0229** |

[1]Numerator degrees of freedom (d.f.) = 1 for each model term; error d.f. = 31

**Supplementary Table 6.** Results of linear models testing variation in the amount of atmospherically derived nitrogen ($^{15}NO_2$-N) incorporated by direct foliar $NO_2$ uptake into *Nicotiana tabacum* leaf tissue, and foliar %N, as a function of low and high $NO_2$ exposure (40ppb vs. 0ppb), herbivory (control vs. damage) and their interaction (experiment 2). Results include a comparison of control plants and damaged plants, using (a) locally damaged ("Local") leaves, (b) undamaged leaves on damaged plants (i.e. systemically induced, "Systemic") and (c) pooled local and systemic leaves. $P \leq 0.05$ in bold.

| | | mg $NO_2$-N assimilation | | | % Foliar N | | |
|---|---|---|---|---|---|---|---|
| Model term | | (a) Local | (b) Systemic | (c) Pooled | (a) Local | (b) Systemic | (c) Pooled |
| $NO_2$ | $\chi^2$ | 30.1732 | 46.2260 | 34.8587 | 3.5794 | 10.9488 | 8.2522 |
| | *P* | **<0.0001** | **<0.0001** | **<0.0001** | **0.0585** | **0.0009** | **0.0041** |
| Herbivory | $\chi^2$ | 4.9133 | 20.1664 | 6.5510 | 0.1140 | 12.3274 | 1.8154 |
| | *P* | **0.0267** | **<0.0001** | **0.0105** | 0.7357 | **0.0004** | 0.1779 |
| $NO_2$ × Herbivory | $\chi^2$ | 3.9834 | 16.7882 | 5.0398 | 1.4477 | 2.7292 | 3.1424 |
| | *P* | **0.0460** | **<0.0001** | **0.0248** | 0.2289 | 0.0985 | 0.0763 |

[1]Numerator degrees of freedom (d.f.) = 1 for each model term; error d.f. = 23

**Supplementary Table 7.** Amounts (mg) of $^{15}NO_2$ derived N (means and SE) in leaf, stem and root tissues in *Nicotiana tabacum* under all combinations of low and high $NO_2$ exposure (40ppb vs. 0ppb), root available $NO_3^-$ (50mM vs. 500mM) and herbivory (presence vs. absence).

| Tissue | $NO_3^-$ | $NO_2$ | Herbivory | Mean | SE |
|---|---|---|---|---|---|
| Leaf | 50mM | 0 | Ctrl | 0.067115 | 0.017108 |
| | | 0 | Dam | 0.050471 | 0.009026 |
| | | 40 | Ctrl | 7.694510 | 2.021862 |
| | | 40 | Dam | 4.287388 | 1.008841 |
| | 500mM | 0 | Ctrl | 0.309888 | 0.088028 |
| | | 0 | Dam | 0.166037 | 0.027076 |
| | | 40 | Ctrl | 12.51712 | 1.384989 |
| | | 40 | Dam | 6.667445 | 0.829721 |
| Root | 50mM | 0 | Ctrl | 0.038073 | 0.009175 |
| | | 0 | Dam | 0.130131 | 0.082201 |
| | | 40 | Ctrl | 1.085739 | 0.236358 |
| | | 40 | Dam | 1.300727 | 0.295260 |
| | 500mM | 0 | Ctrl | 0.032296 | 0.029355 |
| | | 0 | Dam | 0.064490 | 0.044021 |
| | | 40 | Ctrl | 0.536911 | 0.146333 |
| | | 40 | Dam | 0.701396 | 0.075821 |
| Stem | 50mM | 0 | Ctrl | 0.007765 | 0.002932 |
| | | 0 | Dam | 0.011943 | 0.003384 |
| | | 40 | Ctrl | 0.447325 | 0.138017 |
| | | 40 | Dam | 0.876349 | 0.261724 |
| | 500mM | 0 | Ctrl | 0.004240 | 0.002058 |
| | | 0 | Dam | 0.002831 | 0.004517 |
| | | 40 | Ctrl | 1.120962 | 0.346795 |
| | | 40 | Dam | 1.175220 | 0.129116 |
